# Revealing age-related changes of adult hippocampal neurogenesis using mathematical models

**DOI:** 10.1242/dev.153544

**Published:** 2018-01-01

**Authors:** Frederik Ziebell, Sascha Dehler, Ana Martin-Villalba, Anna Marciniak-Czochra

**Affiliations:** 1 Institute of Applied Mathematics, Heidelberg University, Heidelberg 69120, Germany; 2 German Cancer Research Center (DKFZ), Heidelberg 69120, Germany; 3 Interdisciplinary Center of Scientific Computing (IWR) and BIOQUANT, Heidelberg University, Heidelberg 69120, Germany

**Keywords:** Mathematical model, Adult hippocampal neurogenesis, Aging, Stem cells

## Abstract

New neurons are continuously generated in the dentate gyrus of the adult hippocampus. This continuous supply of newborn neurons is important to modulate cognitive functions. Yet the number of newborn neurons declines with age. Increasing Wnt activity upon loss of dickkopf 1 can counteract both the decline of newborn neurons and the age-related cognitive decline. However, the precise cellular changes underlying the age-related decline or its rescue are fundamentally not understood. The present study combines a mathematical model and experimental data to address features controlling neural stem cell (NSC) dynamics. We show that available experimental data fit a model in which quiescent NSCs may either become activated to divide or may undergo depletion events, such as astrocytic transformation and apoptosis. Additionally, we demonstrate that old NSCs remain quiescent longer and have a higher probability of becoming re-activated than depleted. Finally, our model explains that high NSC-Wnt activity leads to longer time in quiescence while enhancing the probability of activation. Altogether, our study shows that modulation of the quiescent state is crucial to regulate the pool of stem cells throughout the life of an animal.

## Abstract (precis)

**Summary:** New deterministic and stochastic mathematical models are proposed to investigate adult neurogenesis in young, old and perturbed hippocampus, and quantified using population-level and clonal experimental data.

## INTRODUCTION

The subgranular zone of the hippocampal dentate gyrus (DG) is one of the two major regions in the adult brain where neural stem cells (NSCs) continuously produce new neurons involved in learning and memory (Ming and Song, 2011). The age-associated impairment of learning and memory has awakened much interest in understanding the cellular and molecular mechanisms underlying the accompanying decline of NSC counts in the hippocampus (Ben Abdallah et al., 2010; Jinno, 2011; Walter et al., 2011; Furutachi et al., 2013; Seib et al., 2013; Jones et al., 2015). Hence, interpretations of the dynamics of NSCs in homeostasis have led to many recent studies (Bonaguidi et al., 2011; Encinas et al., 2011; Mandyam et al., 2007; Sierra et al., 2010; Brandt et al., 2012; Bouchard-Cannon et al., 2013).

To decipher the cellular dynamics behind hippocampal neurogenesis, we apply an interdisciplinary approach combining mathematical modeling with experimental data. These data consist of already published studies (Sierra et al., 2010; Bonaguidi et al., 2011; Encinas et al., 2011; Brandt et al., 2012; Seib et al., 2013) as well as novel data specifically designed to reveal age-related changes during hippocampal neurogenesis. Our approach of combining modeling with data was successfully applied in other stem cell-based systems (Alcolea et al., 2014; Baker et al., 2014; Chabab et al., 2016; Flossdorf et al., 2015; Watson et al., 2015) to identify and quantify stem cell-related processes such as self-renewal and differentiation.

The mathematical model developed describes the evolution of the hierarchical cell production system of adult hippocampal neurogenesis and incorporates basic cell properties, such as quiescence, proliferation, self-renewal and apoptosis. As the configuration of regulatory feedbacks in neurogenesis is not well understood, we follow a top-down approach in which we start with a basic model with constant rates and then apply it to experimental data in order to identify which parameters are changing during aging.

Our study shows that the data are consistent with a model in which NSCs reside in a quiescent phase from which they can either become activated to proliferate or undergo depletion. By applying our model to novel data displaying an age-related accumulation of astrocyte numbers, we demonstrate that about 50% of NSC depletion can be attributed to direct transformation into astrocytes, with the remaining part likely being a result of apoptosis.

Finally, we use our model to uncover the response of the neurogenesis system upon dickkopf 1 (Dkk1) deletion (Seib et al., 2013). We show that the best explanation for the effects upon Dkk1 knockout is that stem cells spend longer time in quiescence but they are also more likely to become activated than being depleted from the quiescent phase.

## RESULTS

### Stem cell model fits population-level and clonal data

**Fig. 1.**
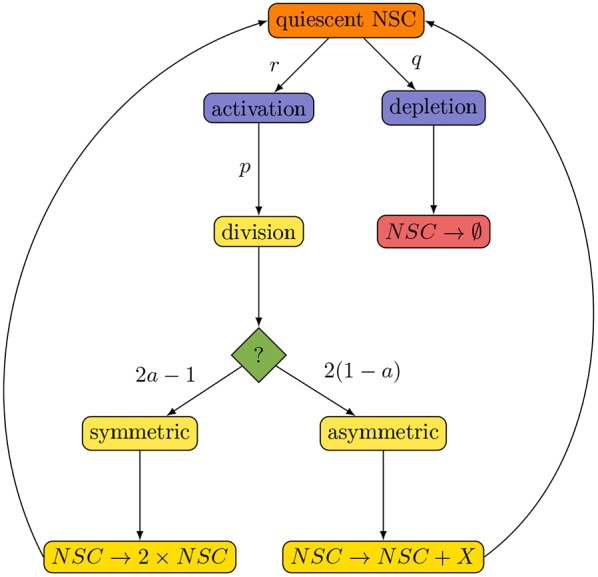
**The proposed model.** Quiescent NSCs are either activated to enter the cell cycle and subsequently perform a symmetric or asymmetric division, or vanish from the NSC pool by a depletion event. Moreover, cycling NSCs re-enter the quiescent phase after division.

Our model is based on a compartment of quiescent NSCs, corresponding to the *G*_0_ phase of the cell cycle (Fig. 1). Those quiescent NSCs have the ability to enter the cell cycle and perform a symmetric self-renewing or asymmetric division, followed by NSCs returning to quiescence after division (Bonaguidi et al., 2011). Although Bonaguidi et al. (2011) do not address a mechanism leading to NSC disappearance, they found an increased number of NSC-depleted stem cell clones in their data and concluded that NSC depletion occurs. Accordingly, we assume an outflow from the quiescent NSCs compartment not only due to cell cycle entry but also due to depletion. As shown later, our results suggest that NSC depletion consists of apoptosis as well as astrocytic transformation. Accordingly, our model is given by the set of equations:
(2.1)
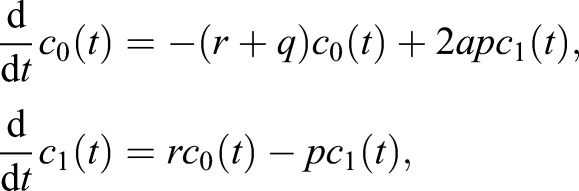
where *c*_0_ represents quiescent NSCs and *c*_1_ cycling NSCs. The parameter *p* denotes the proliferation rate, *q* is the depletion rate and *r* the activation rate (see Fig. 1 for a graphical representation). Moreover, as we consider experimentally observed symmetric and asymmetric NSC divisions (Bonaguidi et al., 2011), we introduce the parameter *a* as the fraction of self-renewal, which is the probability of a progeny cell to have the same fate as the parent cell (Marciniak-Czochra et al., 2009).

**Fig. 2.**
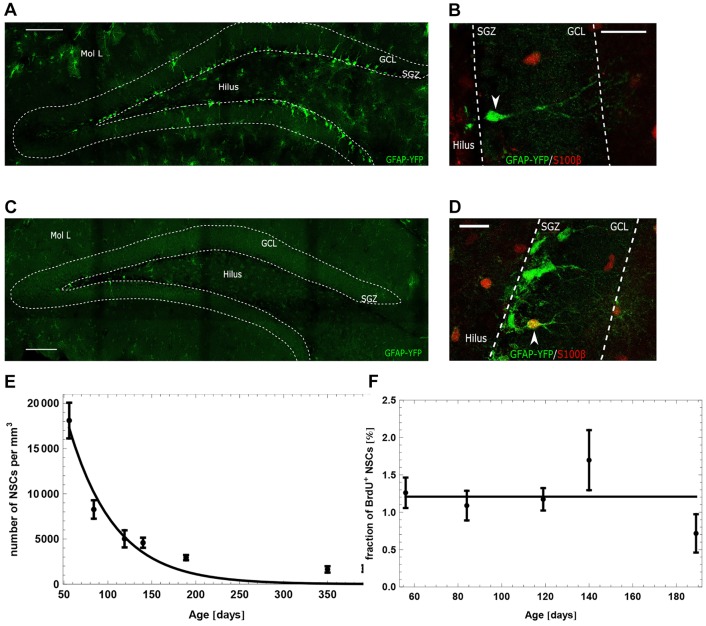
**GFAP-YFP-expressing cells in the DG and population-level dynamics of hippocampal neural stem cells.** (A,C) GFAP-YFP-positive cells in 8-week-old (A) and 56-week-old (C) GFAP-YFP reporter mice. Scale bars: 100 μm. (B,D) Representative confocal images of immunostaining for GFP (green) and S100β (red). Shown are examples of a GFAP^+^/S100β^−^ neural stem cell (B) and a GFAP^+^/S100β^+^ astrocyte (D). Scale bars: 20 μm. (E,F) Fit of the proposed model to the total number of NSCs (E) and the fraction of BrdU-incorporating NSCs (F). Estimated parameters are displayed in Table 1.

We investigate the proposed model by comparison with experimental data. For this, we measure the number of NSCs and the fraction of 5-bromo-2-deoxyuridine (BrdU)-incorporating NSCs at several age points during mouse adulthood (Fig. 2). Our data agree with those reported by Encinas et al. (2011), as we also observed a decline of the NSC pool (Fig. 2E) and a constant fraction of BrdU-incorporating NSCs of ∼1% at all ages (Fig. 2F). By estimating model parameters, we find that the model can be fitted to these population-level data (Fig. 2E,F, black line).

In contrast to the population-level data that account for large cell numbers and admit inferences about the collective behavior of a whole-cell population, clonal data reflect single-cell level behavior by tracking the progeny of individual cells. To assess the clonal dynamics of NSCs, Bonaguidi et al. (2011) labeled individual NSCs at the age of 8-12 weeks and evaluated their clonal progeny 1 month, 2 months and 1 year later. This led to a classification of NSC clones into three categories: quiescent, consisting of exactly one NSC; activated, including one NSC and at least one additional cell; and depleted, containing no NSCs.

**Fig. 3.**
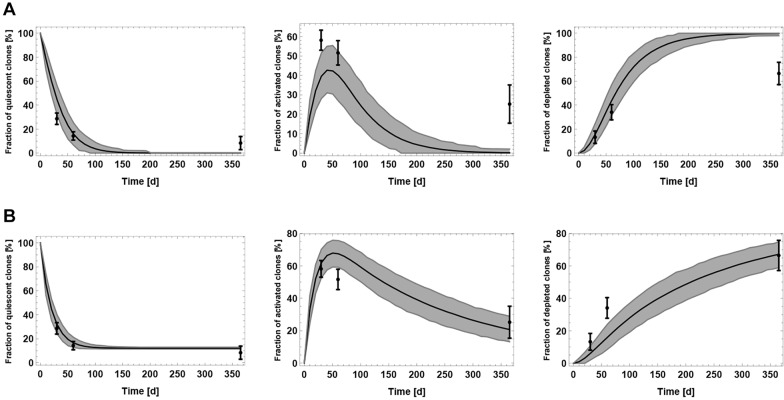
**Comparison of the proposed model with the clonal data of** **Bonaguidi et al. (2011)****.** Results are obtained by simulating 100 NSC clones 1000 times. Simulation data are represented as mean (solid black line) and a band containing 95% (gray) of all simulated trajectories. Black error bars correspond to the clonal data. Estimated parameters are displayed in Table 1. (A) Simulation of the stochastic counterpart of model (2.1) using the parameters of the population-level fit displayed in Fig. 2. (B) Fit of the stochastic counterpart of model (2.1) by estimating model parameters.

While populations of many cells can be modeled using a deterministic approach based on averaging over the population, modeling of clonal data requires a stochastic approach taking into account cellular heterogeneity. Therefore, to fit our model to the clonal data (Fig. 3), we made use of Gillespie algorithm (Gillespie, 1977) to define a stochastic counterpart of model (2.1).

A detailed description of the model quantification procedure is presented in Materials and Methods. Our analysis shows that the population-level and the clonal data set cannot be explained simultaneously and it is necessary to use different parameters to reproduce the two data sets. Simulation of the stochastic version of model (2.1) using the parameters obtained from population-level data does not recapitulate the clonal dynamics data (Fig. 3A), even if one population-level parameter is allowed to be different (Fig. S7). On the other hand, estimating model parameters by fitting the stochastic version of model (2.1) to the clonal data results in a good agreement between observed and predicted clonal behavior (Fig. 3B). However, to obtain a good agreement, it is necessary to assume existence of a population of resilient NSCs. By resilient cells, we mean quiescent NSCs that never become activated or depleted, thus remaining quiescent indefinitely (see ‘Existence of resilient NSCs’ for details). Assuming the existence of such subpopulation prevents a rapid extinction of quiescent clones. According to the model fit, resilient NSCs account for about 10% of all NSCs in a 10-week-old mouse. A model without resilient cells predicts that the fraction of quiescent clones approaches zero, contradicting the clonal data at the 1 year time point. Note that resilient stem cells can be labeled in the clonal experiment of Bonaguidi et al. (2011), because the applied labeling technique does not require cells to divide. Moreover, we want to stress that the concept of resilient stem cells is only needed to fit the clonal data of Bonaguidi et al. (2011), not the population-level data. The discrepancy between clonal and population data may be caused by differences in experimental setup. The differences between the processes quantified by the available data sets may be linked to the sparse labeling protocol in case of the clonal data. It seems to select for a special subset of cells (the ones responding to low doses of tamoxifen). We also note that the clonal data are based on quantification of only about 100 clones and, hence, the parameter estimates are potentially biased.

Model (2.1) assumes that dividing stem cells return to quiescence after division and that depletion events occur from the quiescent phase. An alternative stem cell model has been proposed by Encinas et al. (2011). The latter is based on a hypothesis that stem cell depletion occurs after a series of asymmetric division events. This scenario leads to a mathematical model (Fig. S1) that can be fitted to the population data (Fig. S2) but cannot capture the clonal dynamics (see supplementary information, section: Stem cell depletion coupled to stem cell division; Fig. S3). Hence, in the remainder of this paper we focus on model (2.1).

### Aged stem cells spend longer in quiescence but also have a higher probability of becoming activated

The age-related decline of the NSC pool is a central characteristic of adult hippocampal neurogenesis. We asked whether the decline occurs uniformly with age or if the dynamics of NSCs change during adulthood. In the case of a uniform decline, NSCs numbers are expected to drop exponentially, corresponding to a constant decay rate. As outlined in our previous study (Ziebell et al., 2014), the decline of NSC number saturates with age, resulting in an underestimation of the total number of NSCs in old age (Fig. 2E). This indicates that the dynamics of NSCs change during aging. Using mathematical modeling, we evaluate different scenarios for their ability to explain this saturation.

#### Decreasing depletion

**Fig. 4.**
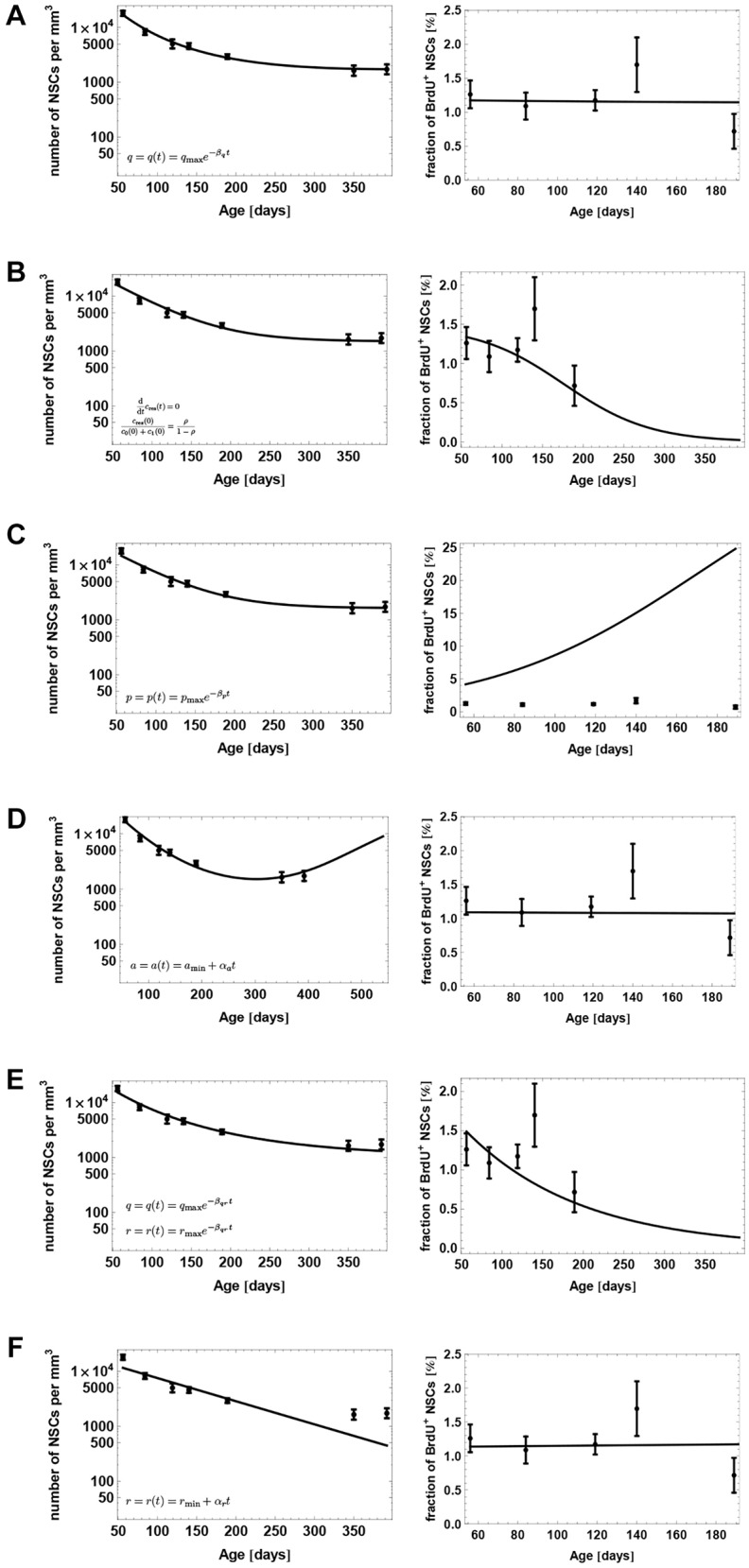
**Evaluation of different scenarios to explain the saturation of the stem cell decline.** Model fit to the population-level data of Fig. 2, assuming (A) a decreasing NSC depletion rate during aging, (B) an additional population of resilient NSCs, (C) age-related lengthening of the cell cycle of NSC, (D) that stem cells increase their self-renewal during aging, (E) that stem cells stay progressively longer in quiescence during aging and (F) that the fraction of activated stem cells increases during aging.

The depletion process is the integral part of the NSC decline. Without it, the number of NSCs would not decrease. One possibility to explain the saturation of decline of NSCs is that the depletion rate of NSCs declines during aging, leading to a decreased fraction of depleting stem cells in old age. Accordingly, we modify model (2.1) by assuming
(2.2)
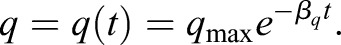
As can be seen from Fig. 4A, the discussed mechanism allows a good fit to the data. The exponential decay function is a particular choice of a time-dependent parameter, reflecting the existence of a feedback loop based on some time-decaying variables. Other plausible control mechanism is a bang-bang control. It can be modeled by a function changing abruptly from a constant positive value to zero. However, assuming a bang-bang decay of the depletion rate does not explain the observed dynamics (data not shown). Our results suggest that the depletion rate is a subject of a nonlinear regulation that might be based, for example, on the total number of stem cells, which is decaying in time.

#### Existence of resilient NSCs

Our analysis of the clonal data set of Bonaguidi et al. (2011) points towards a possible second population of resilient NSCs: cells that can neither become activated nor depleted. The additional population *c*_res_ is implemented with

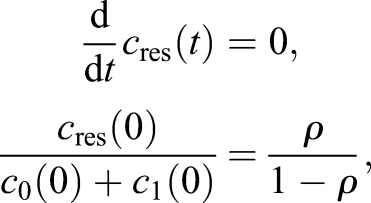
where *ρ* is the fraction of resilient NSCs of all NSCs at the begining of adulthood. However, existence of such a population in combination with the decline of NSCs leads to a decrease of the fraction of cycling NSCs (Fig. 4B), contradicting the observed constant fraction of BrdU-labeled NSCs in old age (Encinas et al., 2011).

#### Lengthening of the cell cycle

If NSCs take progressively longer during aging to complete the cell cycle, the number of stem cells entering the quiescent phase declines with time, leading to a decreasing net depletion of stem cells residing in quiescence. Accordingly, we assume a decline of the proliferation rate given by

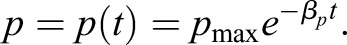
A comparison of the suggested mechanism with the data shows a lack of fit, because lengthening the cell cycle dramatically increases the fraction of cycling NSCs and as a result the fraction of BrdU-incorporating NSCs (Fig. 4C).

#### Increasing self-renewal

The saturation of the NSC decline could also indicate that NSCs increase their self-renewal to counteract the depletion. The corresponding modification takes the form

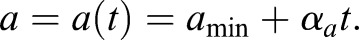
An analysis of this scenario shows that, in order to fit our population level data, NSC number should increase at the age of about 1 year (Fig. 4D). However, this implication contradicts the fact that NSCs and other downstream compartments, such as neural progenitors and immature neurons, decline in number even at later time points than 1 year of age (Encinas et al., 2011; Walter et al., 2011).

#### Increasing quiescence

An increase in NSC quiescence, corresponding to an age-related lengthening of the *G*_0_ phase, could also explain the decline pattern of NSCs. Because leaving the quiescent phase is associated with a higher tendency to deplete than to maintain or expand the pool of NSCs – otherwise it could not be explained why this pool declines – remaining in quiescence would neutralize the decline. The modification takes the form

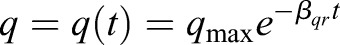
and

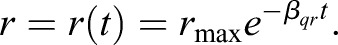
The justification for these equations is that leaving the quiescent phase *c*_0_ is driven by a joint decay process (Magill and Galy, 2005), which consists of activation (with rate *r*) and depletion (with rate *q*). The mean time of NSCs to sojourn in quiescence is thus given by

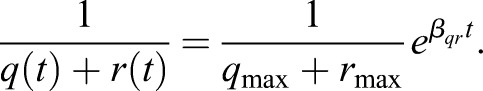
However, in order to explain the saturation of NSC decline in this scenario, the strong increase of quiescence reduces the fraction of cycling NSCs (Fig. 4E), contradicting the observation of a constant fraction of BrdU-incorporating stem cells up to even 20 months of age (Encinas et al., 2011).

#### Increasing activation

Another possible explanation for the saturation of the NSC decline is that the fraction of quiescent NSCs that becomes activated per time unit increases during aging. As activation is associated with maintenance or expansion of the NSC pool, increasing activation could counteract the NSC decline in old age. The increasing activation scenario is implemented with

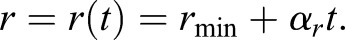
However, comparing the above mechanism with our population level data shows that an increase of activation fails to explain the saturation of NSC decline (Fig. 4F).

#### Model selection

In conclusion, the only biologically plausible scenario reached by visually comparing the model fit to the data is that of a decreasing depletion rate. To also evaluate each of the above model modifications from a statistical perspective, we compute for each scenario the corresponding Akaike information criterion (AIC) score. This model selection score quantifies the trade off between the complexity of a model, i.e. number of parameters, and the goodness of fit to the data (Burnham and Anderson, 2002). Using this score, the only considerable mechanisms besides the decreasing depletion mechanism are the scenario of increasing self-renewal and the existence of a population of resilient NSCs (see Materials and Methods). However, as outlined previously, the former scenario contradicts the observation of a decline of NSCs and downstream cell types in old age (Walter et al., 2011), whereas the latter contradicts the observation of a constant fraction of BrdU-incorporating NSCs up to 20 months of age (Encinas et al., 2011). These considerations lead to the conclusion that, among the contemplated mechanisms, the decreasing depletion scenario is the only plausible explanation for the saturation pattern of the NSC decline.

#### Biological interpretation

**Fig. 5.**
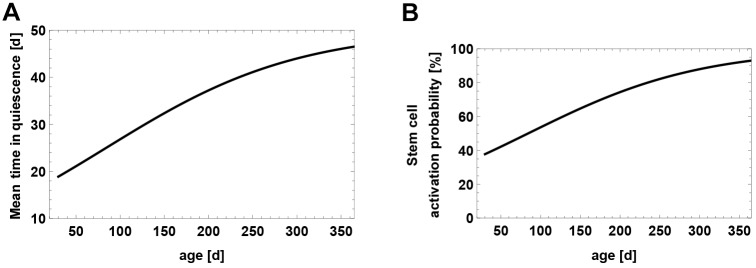
**Predicted age-related changes.** (A) The average time a stem cell stays in the quiescent phase and (B) the probability a stem cell becomes activated rather than depleted from the quiescent phase.

Only a decreased depletion rate of NSCs can reproduce these data without making biologically implausible predictions. A further quantification of this scenario suggests that aged stem cells stay longer in the quiescent stage (Fig. 5A), but also have a higher probability of reactivation (Fig. 5B). Although the two statements may seem contradictory, our model assumes that leaving quiescence can be achieved through activation or depletion. Thus, the latter assertion merely states that the probability of exiting the quiescent stage through activation versus depletion increases during aging.

### Stem cell depletion is driven by astrocytic transformation and apoptosis

**Fig. 6.**
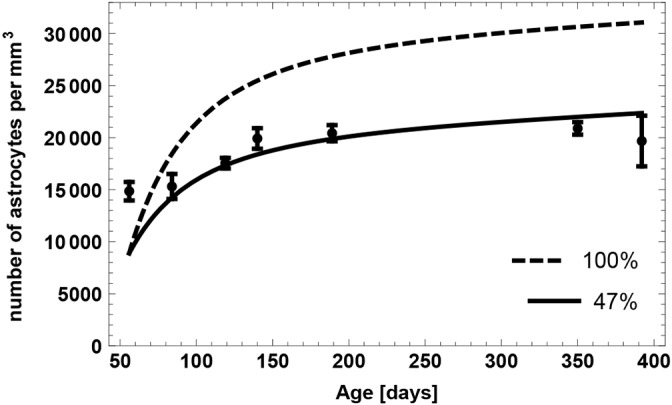
**Age-related accumulation of astrocyte numbers.** Dashed line represents expected number of astrocytes, assuming that 100% of the NSC decline is caused by astrocytic transformation. Solid line results from fitting the fraction of transformation events to the data, indicating that about half of the NSC decline is caused by astrocytic transformation.

**Fig. 7.**
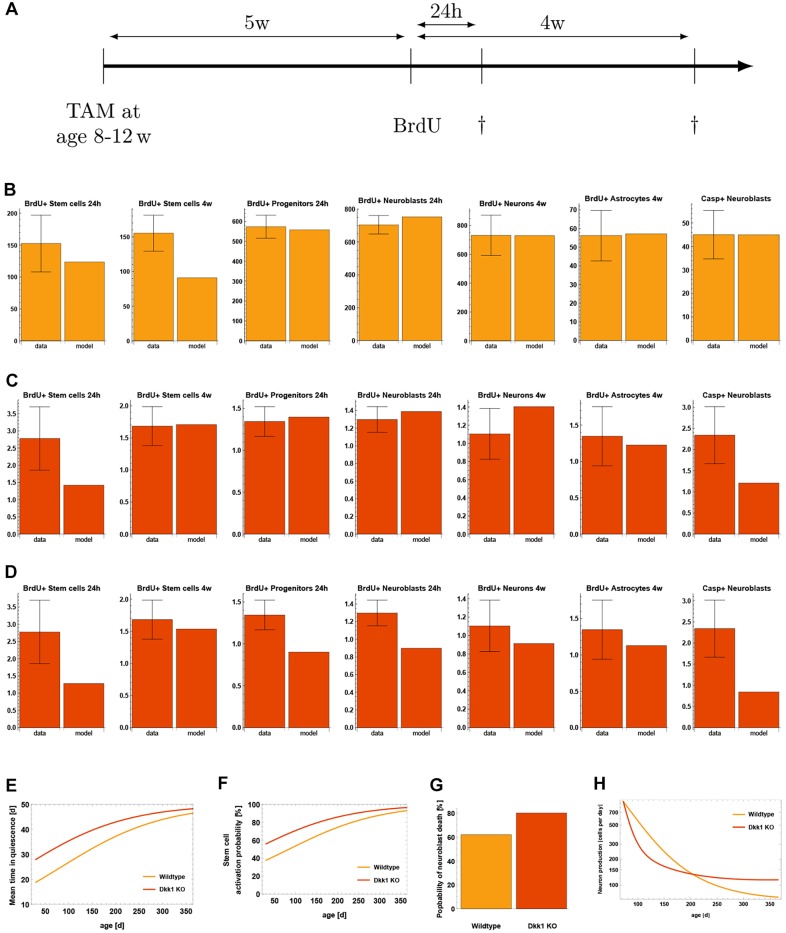
**Evaluation of the Dkk1 knockout study of** **Seib et al. (2013****).** (A) The experimental protocol. (B) Comparison of the wild-type part of the data and the mathematical model. (C) Observed KO effects versus model, assuming decreased NSC depletion rate. KO effects are defined as ratio of the KO data to the corresponding wild-type data, i.e. KOeffect=(KOdata)/(WTdata). (D) Observed KO effects versus the model, assuming increased NSC self-renewal. (E) Mean time of a stem cell to remain in the quiescent stage. In the case of Dkk1 deletion, this time is increased. (F) Age-dependent activation probability of stem cells. In the KO case, NSCs have a higher activation probability. (G) Probability of a neuroblast dying rather than maturing into a neuron. In the case of Dkk1 knockout, a higher fraction of neuroblasts die. (H) Production rate of new neurons. Dkk1 deletion alters the number of newborn neurons and results in higher neuron production in old age.

In order to explain the decline of NSC numbers during aging, Encinas et al. (2011) suggested that NSCs deplete by transforming into astrocytes. To test this theory, we counted the total number of astrocytes during aging to see whether astrocytes accumulate in the DG due to stem cell depletion. We also checked whether newborn astrocytes migrate away from the granule cell layer and escape counting. To achieve this, we used sparse labeling of Nestin-YFP reporter mice that exclusively marked NSCs and their progeny upon tamoxifen injection within the subgranular zone. We did not observe any YFP-positive cells outside the dentate gyrus 90 days after the last tamoxifen injection. As we find an increasing progression of the astrocyte count (Fig. 6), we use our model to test whether the increase in astrocyte numbers corresponds to the observed stem cell decline. For this, we take model (2.1) together with our best explanation of the saturation of the NSC decline (2.2) and add a new compartment, *c*_2_, of astrocytes satisfying
(2.3)
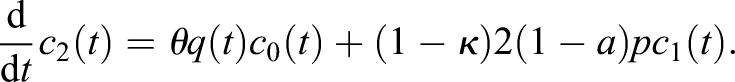
Here, *θ*∈[0, 1] is the fraction of NSC depletion events where depletion occurs via astrocytic transformation. Although the first term in the above sum accounts for transformation of stem cells into astrocytes, the second term models the number of astrocytes being produced from stem cells through asymmetric divisions. Both parameters, *θ* and *κ*, are estimated by fitting the astrocyte data displayed in Fig. 6 and wild-type neurogenesis data of a Dkk1 knockout study (Fig. 7B) laid out in the next section.

Estimating the transforming fraction *θ* yields *θ*=0.473. Thus, modeling reveals that astrocytic transformation only partially contributes to about half of the stem cell decline. If every NSC (100%) depletes through transformation into astrocytes, the final astrocytic yield would be much higher than what is observed (Fig. 6). The remaining 53% of the NSC decline are likely a result of apoptosis and further modeling laid out in the supplementary information (section: Astrocytic transformation) shows that NSC apoptosis is almost non-detectable due to rapid phagocytosis by microglia (Sierra et al., 2010).

It is also possible that the accumulation of astrocyte numbers can be explained with a higher transformation probability, *θ*, if additionally astrocytes are allowed to undergo apoptosis, thus counteracting the total astrocytic yield. We also analyzed this scenario in the supplementary information (section: Astrocytic transformation) and find that apoptosis cannot account for a higher transformation probability.

### Upon Dkk1 deletion, stem cells spend longer in quiescence but are more likely to become re-activated

We apply our quantified model of NSC dynamics to the study of Seib et al. (2013), in which the Wnt antagonist dickkopf 1 (Dkk1) was deleted in NSCs. The deletion led to a larger NSC pool that even counteracted the age-related decline (Seib et al., 2013). In view of published studies (Qu et al., 2010; Munji et al., 2011), we previously hypothesized that NSC numbers expand by increasing the fraction of self-renewal in response to increased Wnt activity. Now, we used the developed model to identify the most likely change in cellular dynamics that explains Wnt-induced expansion of the pool of NSCs.

First, we extend our stem cell model (2.1), including aging effects (2.2) and astrocytic transformation (2.3), by adding all cell types necessary to simulate hippocampal neurogenesis. In particular, we assume that: (1) NSCs generate progenitors and astrocytes via asymmetric divisions (Bonaguidi et al., 2011); (2) progenitors perform a sequence of symmetric divisions followed by differentiation into neuroblasts (Encinas et al., 2011); and (3) neuroblasts undergo apoptosis as well as neuronal differentiation (Sierra et al., 2010). Thus, the full model of wild-type neurogenesis is given by the set of equations:
(2.4)
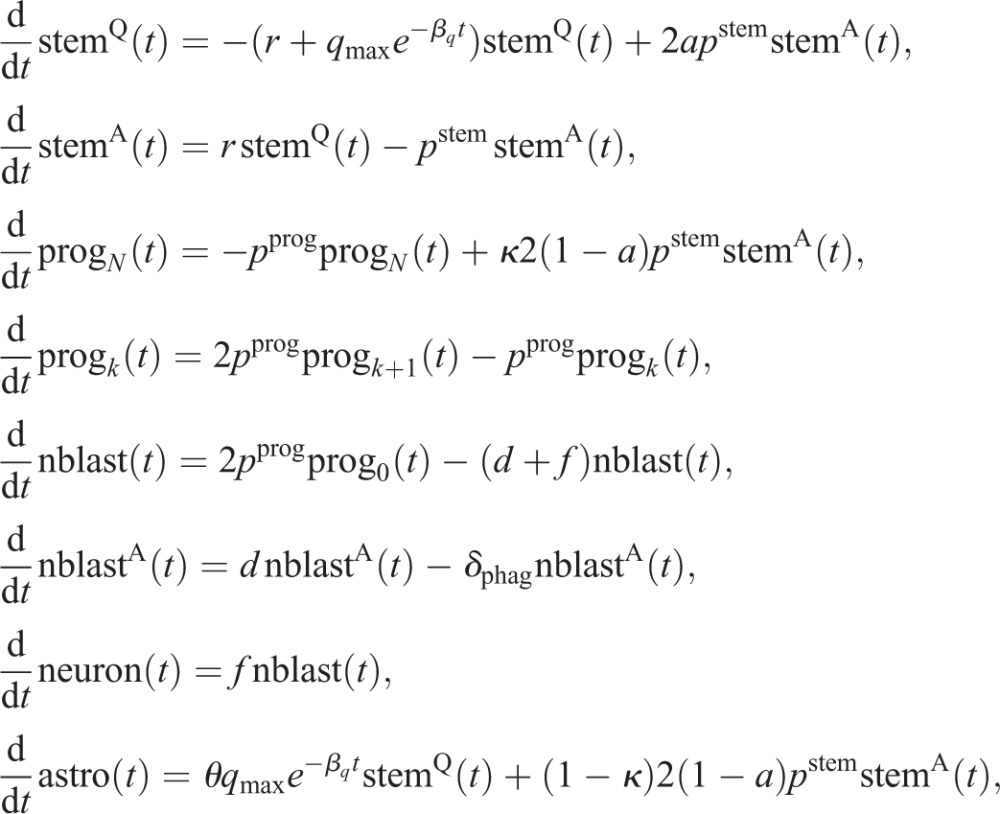
where stem^Q^ denotes quiescent stem cells, stem^A^ active (cycling) stem cells, prog_*i*_ indicates progenitors with *i* remaining divisions (1≤*i*≤*N*), nblast indicates neuroblasts, nblast^A^ indicates apoptotic neuroblasts, neuron indicates mature neurons and astro indicates astrocytes.

Next, we used the full model to simulate the protocol of the Dkk1 study (Fig. 7A). In the experiment, adult mice were injected with tamoxifen (TAM) to induce deletion of Dkk1. Following a 5-week period, BrdU was administered and chased for 24 h and 4 weeks. The number of BrdU-labeled cells among the different populations following Dkk1 deletion was quantified and compared with numbers in wild-type animals.

In order to explain the effects of Dkk1 deletion, we first reproduced the wild-type part of the data using our model. As can be seen from Fig. 7B, the model displays a very good fit to the wild-type data. Interestingly, a prediction of this fit is that 38% of the neuroblasts mature into neurons and about 62% undergo apoptosis. This finding is consistent with experimental observations stating that the major part of immature neurogenic progenitors undergoes apoptosis rather than differentiating into neurons (Sierra et al., 2010), thus further supporting our modeling approach.

We then focus on explaining the effects of Dkk1 knockout (KO). To model the effects of Dkk1 deletion, we introduced additional parameters into the wild-type model (2.4). As Dkk1 was selectively deleted in NSCs, we assume that knockout effects were the result of changes in the value of stem cell parameters. We thus consider the parameters *a*, *p*^stem^, *q*_max_ and *r* as possible candidates for being changed upon Dkk1 deletion. In addition, the data indicate an increased death rate *d* of neuroblasts after the knockout. For each candidate parameter

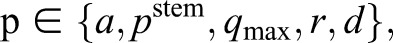
we introduce a change Δ_**p**_ such that the corresponding wild-type and knockout parameters are related via

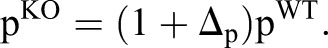
Hence, Δ_**p**_ denotes the relative change of the wild-type parameter p. Moreover, as the model fit to the wild-type part of the data is not perfect, instead of considering the KO data we consider KO effects, defined as the ratio of the KO data to the corresponding wild-type (WT) data, i.e. KO effect = (KO data)/(WT data).

We then apply statistical measures to analyze different combinations of the candidate parameters for their plausibility of being altered simultaneously by Dkk1 deletion using AIC scores. We find that the most plausible explanation of the observed changes is a decreased depletion rate *q*_max_ in combination with increased neuroblast apoptosis *d* (Fig. 7C). In contrast, statistical analysis shows that alternative scenarios such as increased NSC self-renewal (Fig. 7D) can be discarded as explanation of the KO data (see Materials and Methods). To quantify the above mentioned explanation of decreased NSC depletion rate *q*_max_ and increased neuroblast apoptosis rate *d*, we calculate the average time a stem cell remains quiescent (Fig. 7E) and the probability that leaving quiescence is driven by activation, i.e. cell cycle entry, rather than depletion (Fig. 7F). In young individuals, stem cells remain quiescent for about 10 days longer and have a 20 percentage points increased activation probability in the case of Dkk1 deletion, whereas those differences diminish during aging. Moreover, Dkk1 knockout increases the probability that neuroblasts undergo apoptosis rather than neuronal differentiation from about 60% to about 80% (Fig. 7G). Interestingly, Dkk1 deletion impacts the production rate of new neurons (Fig. 7H). In young individuals, neuron production is decreased due to increased NSC quiescence. The latter leads to increased NSC numbers in old individuals that, in turn, results in increased neuron production.

## DISCUSSION

To obtain insights into the control mechanisms of stem cell dynamics and age-related effects during hippocampal neurogenesis, we have applied an interdisciplinary approach based on mathematical modeling and experimental data. This approach allows investigating normal and perturbed neurogenesis by quantifying cell dynamics that cannot be measured experimentally.

Our stem cell model (2.1) was motivated by the experimental observations of Bonaguidi et al. (2011) and subsequently extended combining data on NSCs cell cycle properties (Brandt et al., 2012), astrocytic transformation (newly generated), progenitor dynamics (Encinas et al., 2011) as well as neuroblast dynamics and phagocytosis (Sierra et al., 2010). Thus, our modeling framework integrates data describing neurogenesis on different levels in a coherent manner.

Although the original stem cell model (2.1) can reproduce the available population-level and clonal data, it should be noted that both data sets reflect different dynamics corresponding to different cell parameters. There might be several reasons for such discrepancy. Different morphological and genetic features used to define NSCs may correspond to distinct subpopulations of stem cells or, conversely, to slightly different differentiation stages within a continuum between a stem cell and a neural progenitor. In addition, the clonal data account for relatively few cells collected from multiple individuals. Thus, averaged clonal data, as in the case of Bonaguidi et al. (2011), could be biased due to biological variability among animals or due to certain cell fate choices that stochastically dominate due to the paucity of analyzed clones.

We next addressed possible mechanisms that can explain the saturation pattern of the age-related decline in NSC numbers – an observation that confirms previous data by Encinas et al. (2011). After evaluating several stem-cell features, the only explanation left is that stem cells spend progressively longer in quiescence during aging. At the same time, a greater fraction of aged NSCs becomes re-activated from the stem cell pool rather than being depleted. Our reasoning is coherent from a modeling perspective, as we have ruled out several alternative scenarios.

Another issue related to age-related changes during hippocampal neurogenesis is that of the mechanism leading to the decline of NSC numbers. Here, we were able to partially confirm the astrocytic transformation hypothesis of Encinas et al. (2011) by observing an age-related accumulation of astrocyte numbers. Mathematical modeling revealed that these transformation events account for about half of the NSC decline. Interestingly, our calculations showed that even if the remaining half of the decline is caused by NSC apoptosis, detection of apoptotic cells is not possible due to rapid phagocytosis (Sierra et al., 2010). Similarly high estimates of apoptosis have been obtained in a modeling study of neurogenesis based on a stochastic branching process approach (Li et al., 2017).

One of the main aims of mathematical modeling is to develop model-based predictions that can be tested experimentally. Such model validation is an important step within the scientific method (Popper, 1959). We thus applied our model to the study of Seib et al. (2013), in which the Wnt inhibitor Dkk1 was deleted. The very good fit to the wild-type data, which results from accurate simulation of the experimental protocol, confirms that our model accounts for key aspects of hippocampal neurogenesis. Moreover, the fitting procedure has led to the prediction that the majority of neuroblasts undergoes apoptosis instead of neuronal differentiation, a prediction that has already been validated (Sierra et al., 2010). Moreover, we have analyzed multiple scenarios to reveal the effects of Dkk1 deletion. Originally, Seib et al. (2013) hypothesized that Dkk1 knockout increases the self-renewal of NSCs. This reasoning was based on previous studies showing that Wnt signaling induces self-renewal of radial glia progenitors in the embryonic brain (Munji et al., 2011) and that Wnt ligands were reported to increase the proliferation and self-renewal of NSCs (Gao et al., 2007; Lie et al., 2005; Michaelidis and Lie, 2008; Qu et al., 2010). Importantly, the self-renewal and its regulation has been also suggested by mathematical models in context of clonal competition, selection and emergence of resistance in cancer cell populations (see Stiehl and Marciniak-Czochra, 2017). However, our calibrated models of neurogenesis suggest that the only considerable scenario is that of an increased NSC quiescence in conjunction with an increased probability to exit the quiescent phase through activation rather than depletion. In contrast, increased self-renewal of NSCs can not explain the effects upon Dkk1 knockout. The reason for this is that increasing the self-renewal of NSCs would increase the fraction of quiescent NSCs present in the NSC pool, as our model assumes NSCs return to quiescence after division (Bonaguidi et al., 2011). Together with this, a decreased fraction of BrdU-positive downstream cell compartments would be observed. Taken together, our results indicate that regulating the rate of activation versus depletion into astrocytes seems to represent a central hub to fine-tune neurogenesis.

Our study shows that mathematical modeling is a powerful tool to investigate complex cell systems such as the neurogenic niche of the hippocampus. It is possible to describe the dynamics of stem cell systems using both deterministic (Enderling et al., 2007; Marciniak-Czochra et al., 2009; Stiehl and Marciniak-Czochra, 2011; Stiehl et al., 2014; Weekes et al., 2014) and stochastic models (Klein et al., 2010; Doupé et al., 2012; Chabab et al., 2016). Although the former usually have the form of linear or nonlinear differential equations, the latter are of at least two possible kinds. One is the system of master equations (Chapman–Kolmogorov forward equations if the model is Markovian), which allow computing probability distributions of the state variables as a function of time (Frede et al., 2016). If it is desirable to obtain longitudinal information, i.e. information concerning the possible time trajectories of the process, the most common practical solution is simulation. A fusion of deterministic and stochastic simulation approaches, as conducted in this study by employing the Gillespie algorithm (Gillespie, 1977), is very effective at integrating population-level and single cell-level data. The initial deterministic stem cell model consists of linear differential equations featuring constant rates. Such linear models have been successfully applied before to processes close to homeostasis (Busch et al., 2015) or uncontrolled growth in cancer (Michor et al., 2005; Weekes et al., 2014). In our case, using a linear model allowed the identification of time-dependent parameters that are necessary to explain age-related changes during neurogenesis. This, in turn, suggests which parameters are subject to a nonlinear regulation.

Following a parsimonious approach to modeling in which comprehensive models are better understood in view of simpler models, we have always departed from a minimal set of the processes and extended the models upon their verification using different independent data sets. In this way we have learnt which assumptions are needed to explain different observations. Certainly, there exist different aspects of neurogenesis that have not been considered in the model so far. Among others, our model does not explicitly include the historically verified population of Sox2^+^ early intermediate progenitor cells (eIPCs; e.g. Encinas et al., 2006; Suh et al., 2007) as a separate component. The minimum set of assumptions that were used to build the present model include the population of eIPCs that are nestin, Sox2 and Tbr2 positive (type 2b only) with the type2a of nestin/Sox2-positive and Tbr2-negative cells included within the NSC population. A model including all these subpopulations as separate compartments will be much more complicated. Another limitation of the present model is that it divides stem cells into two compartments, quiescent and active, whereas in reality a continuous spectrum of stem cell activity might be present. Extensions of the model to account for heterogeneity of different cell subpopulations will increase the degree of freedom (number of parameters). Testing such models will require more resolution of the quantitative data and specific hypotheses concerning the impact of the inter-population heterogeneity on the observed dynamics of the whole system.

## MATERIALS AND METHODS

### Animals

GFAP-CreER^T2^-YFP and Nestin-CreER^T2^-YFP reporter mice were housed in the animal facilities of the German Cancer Research Center (DKFZ) in a 12 h dark/light cycle with free access to food and water. All animal experiments were performed in accordance with the institutional guidelines of the DKFZ and were approved by the Regierungspräsidium Karlsruhe, Germany.

### Tamoxifen and BrdU administration

GFAP-CreER^T2^-YFP mice received an intraperitoneal (i.p.) tamoxifen (Sigma; T5648) injection (40 mg/kg) twice a day (morning and evening) for five consecutive days. Two days after tamoxifen treatment, a single shot of BrdU (Sigma; B5002; 150 mg/kg) was injected intraperitoneally and mice were sacrificed 2 h later.

### Long-term tracing of NSC-derived astrocytes

Nestin-CreER^T2^-YFP reporter mice received tamoxifen injections as described above and were sacrificed 90 days after the last injection. Thereafter, the presence of YFP-labeled cells outside the hippocampal region was examined. We did not find any YFP-positive cells outside the DG granule cell layer.

### Tissue preparation and staining

Animals were perfused with 1× Hanks’ Balanced Salt Solution (HBSS) (Gibco; 14170-088) and 4% paraformaldehyde (PFA) (Carl Roth; P087.1). Subsequently, the brain was fixed overnight in 4% PFA and coronal brain slices were cut at 50 μm using a Leica VT 1200S vibratome.

### Immunohistochemistry

For each animal, six 50 μm coronal brain sections (250 μm between sections) were washed four times, for 10 min in Tris-buffered saline pH 7.4 (TBS) at room temperature, blocked for 1 h in TBS containing 3% horse serum (Millipore; S9135) and 0.3% Triton X-100 (Sigma; 9002-93-1) at room temperature, followed by an overnight staining at 4°C in TBS containing 3% horse serum and 0.3% Triton X-100 with the following primary antibodies: rat anti-BrdU (Abcam, ab6326; 1/200), chicken anti-GFP (Aves, GFP-1020; 1/500), mouse anti-S100β (Abcam, ab66028; 1/100) and rabbit anti-Tbr2 (Abcam, ab23345; 1/300). The next day, brain sections were washed four times for 10 min in TBS at room temperature, blocked for 30 min TBS containing 3% horse serum and 0.3% Triton X-100 at room temperature, and stained for 2 h in TBS containing 3% horse serum and 0.3% Triton X-100 at room temperature with the following secondary antibodies: donkey anti-rat 405 (Abcam, ab175670; 1/400), donkey anti-chicken 488 (Jackson ImmunoResearch, 703-545-155; 1/400), donkey anti-mouse 549 (Jackson ImmunoResearch, 715-507-003; 1/400) and donkey anti-rabbit 647 (Jackson ImmunoResearch, 711-605-152; 1/400). Afterwards, sections were washed four times for 10 min in TBS at room temperature and mounted on glass slides.

### Imaging and quantification

For each dentate gyrus, confocal *z*-stacks (2 μm between images) were acquired with a Leica TCS-SP5 confocal microscope with a 20× oil immersion objective and a resolution of 1024×1024 at 100 Hz. Obtained images were analyzed using the ImageJ Cell Counter Plugin to manually count and mark single cells of different cell types. Different cell populations were defined using the following marker combinations: neural stem cells (YFP^+^/S100β^−^/Tbr2^–^), astrocytes (S100β^+^/Tbr2^–^) and cycling cells that gained the marker BrdU by retaining their markers described above. Determined cell numbers were quantified as number of cells per mm^3^ in the DG granule cell layer. The DG volume was calculated by the area of the DG on the central image of the *z*-stack (measured with ImageJ) multiplied with the *z*-stack size.

### Mathematical modeling

#### Quantification

The stem cell model (2.1) contains four parameters: *p*, *q*, *r* and *a*. The value of the proliferation rate *p* can be inferred from the literature (Brandt et al., 2012), as the length of the cell cycle of NSCs was measured as

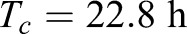
and we can interpret this value as the doubling-time of an exponential growth process, leading to

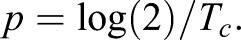
To establish a regime of admissible values of the fraction of self-renewal *a*, we notice that in our model the probability of a symmetric cell division is equal to 2*a*−1 and the probability of an asymmetric cell division is 2(1−*a*) (as denoted on the arrows in Fig. 1) (Ziebell et al., 2014).

To show it, we introduce a new variable *s* denoting the probability of a symmetric cell division. Hence, the probability of an asymmetric cell division is equal to 1−*s*. Probability of a progeny cell to have the same fate as the parent cell can be calculated as *a*=*s*+((1−*s*)/2), i.e. the probability that there is a symmetric cell division *s* multiplied by the probability (equal to 1) that a cell arising in such a division is a stem cell, plus the probability that there is an asymmetric cell division 1−*s* multiplied by the probability that a cell arising at such division is a stem cell. The latter is equal to 1/2, because it is one out of two cells arising from an asymmetric cell division. Hence, we obtain *a*=*s*+((1−*s*)/2)=((1+*s*)/2). Inverting this formula, we obtain *s*=2*a*−1. Consequently, the probability of an asymmetric cell division is equal to 1−*s*=2(1−*a*).

Following the suggestion of Bonaguidi et al. (2011) that the fraction of symmetric NSC divisions is relatively small, we assume 2*a*−1=5%, which yields *a*=0.525 to be an upper limit for the fraction of symmetric NSC divisions. Numerical simulations indicate that increasing values of *a* up to *a*=0.6 (corresponding to 20% of NSC symmetric divisions) do not affect model conclusions (see the supplementary information, section: Sensitivity analysis; Table S1).

The remaining two free parameters *q* and *r* are estimated from the population-level data of the total number of stem cells (Fig. 2E) and the fraction of BrdU-incorporating stem cells (Fig. 2F). To fit the BrdU incorporation data, we assume that the fraction of BrdU-incorporating NSCs is the product of the fraction of cycling NSCs out of all NSCs multiplied by the relative length of the S-phase in the cell cycle, *T*_*s*_/*T*_*c*_, with 
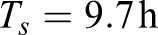
 (Brandt et al., 2012). The reason is that the thymidine analog BrdU can only be incorporated in cells that are at the stage of DNA synthesis.

In the case of estimating *q* and *r* from the clonal data (Fig. 3), the model was converted into a corresponding stochastic processes using the Gillespie method (Gillespie, 1977). Afterwards, 10^4^ NSC clones were simulated and the resulting mean fraction of quiescent, activated and depleted clones was fitted to the clonal data. The Gillespie method allows the simulation of stochastic trajectories of the system with mean values at each time step satisfying the corresponding differential equation. Accordingly, the set of reactions used to simulate the clonal data is given by

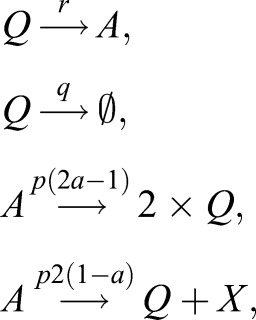
where *Q* denotes quiescent stem cells, *A* active stem cells, 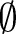 denotes depletion of stem cells from the stem cell pool and *X* denotes a non-stem cell (Fig. 1). To incorporate resilient NSCs into this model, we assume that a fraction *ρ* of all simulated quiescent NSCs does not change, whereas only a fraction, 1−*ρ*, of those cells is subject to the first two reactions.

Model fitting was carried out using the *NonlinearModelFit* procedure of Mathematica 9 (Wolfram Research) to numerically minimize the weighted sum of squared residuals. Weights were chosen as inverse squares of the s.e.m. of the data points according to the Mathematica documentation for fitting data with measurement errors. Estimated parameters for the stem cell model are summarized in Table 1.

**Table 1.**
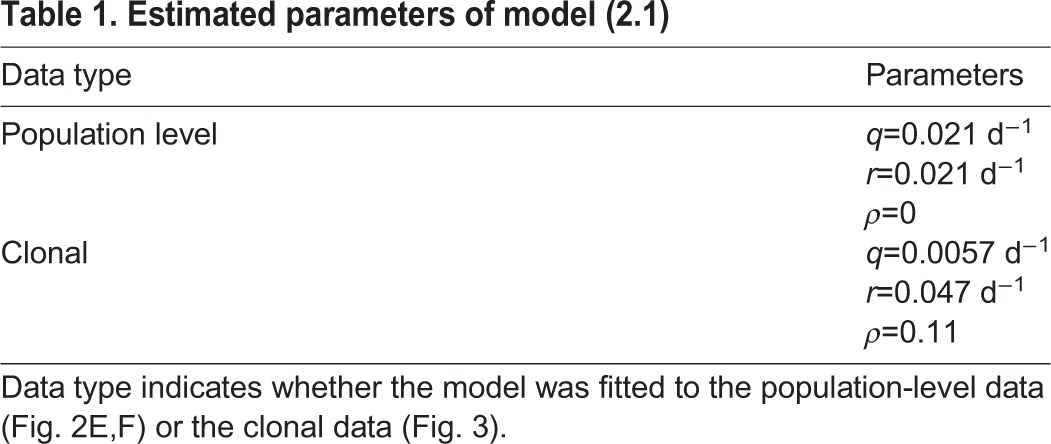
**Estimated parameters of model (2.1)**

The full neurogenesis model (2.4) contains 12 parameters, of which eight are already estimated or taken from the literature (Table 2). In particular, the parameters corresponding to progenitor cells were fitted to a time course of BrdU-labeled progenitors (Figs S4, S5). The parameters *κ*, *d* and *f* are not known prior to the Dkk1 experiment and need to be inferred from fitting the full model to the astrocyte accumulation data (Fig. 6) and the wild-type part of the Dkk1 data (Fig. 7B).

**Table 2.**
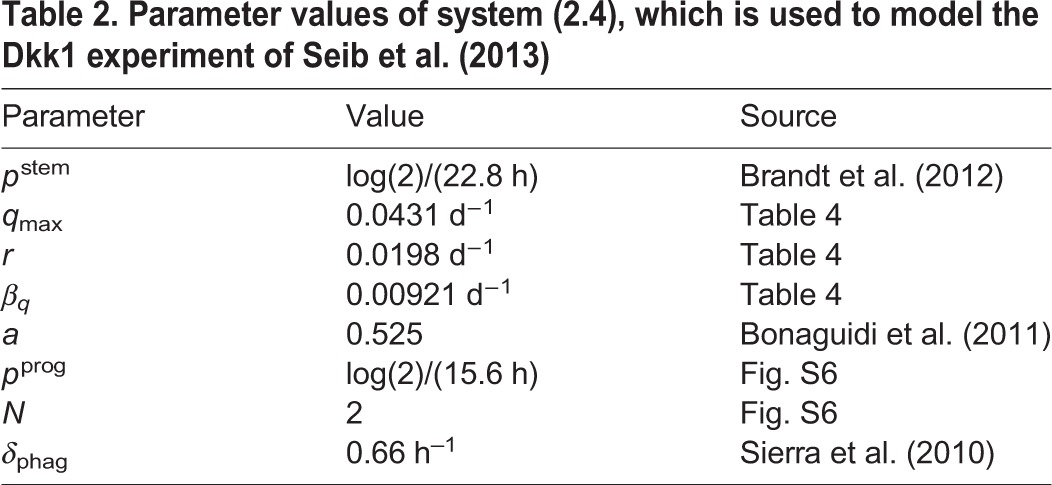
**Parameter values of system (2.4), which is used to model the Dkk1 experiment of** **Seib et al. (2013)**

Estimating the unknown parameters *θ*, *κ*, *d* and *f* leads to the values displayed in Table 3. As previously stated, the resulting fraction of surviving neuroblasts,

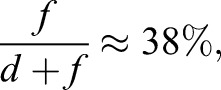
is consistent with experimental findings (Sierra et al., 2010).

**Table 3.**
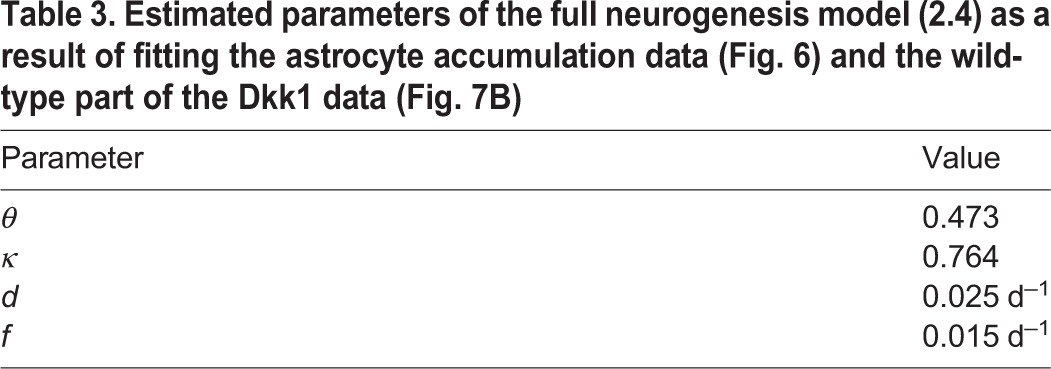
**Estimated parameters of the full neurogenesis model (2.4) as a result of fitting the astrocyte accumulation data (****Fig. 6****) and the wild-type part of the Dkk1 data (****Fig. 7****B)**

#### Model selection

To assess the plausibility of the different scenarios to explain the saturation of the NSC decline, we make use of model selection theory. For each scenario, we compute its corresponding AIC value and compare the resulting Akaike weights Δ (Table 4), which penalize overly complex models. The recommendation is that the level of empirical support of a certain model is substantial if 0≤Δ≤2, considerably less if 4≤Δ≤7 and essentially none if Δ>10 holds (Burnham and Anderson, 2002). Thus, the only considerable mechanisms besides the decreasing depletion mechanism are the scenario of increasing self-renewal and the existence of a population of resilient NSCs (Table 4). However, as outlined previously, the former scenario contradicts the observation of a decline in NSCs and downstream cell types in old age (Walter et al., 2011), whereas the latter contradicts the observation of a constant fraction of BrdU-incorporating NSCs up to 20 months of age (Encinas et al., 2011).

**Table 4.**
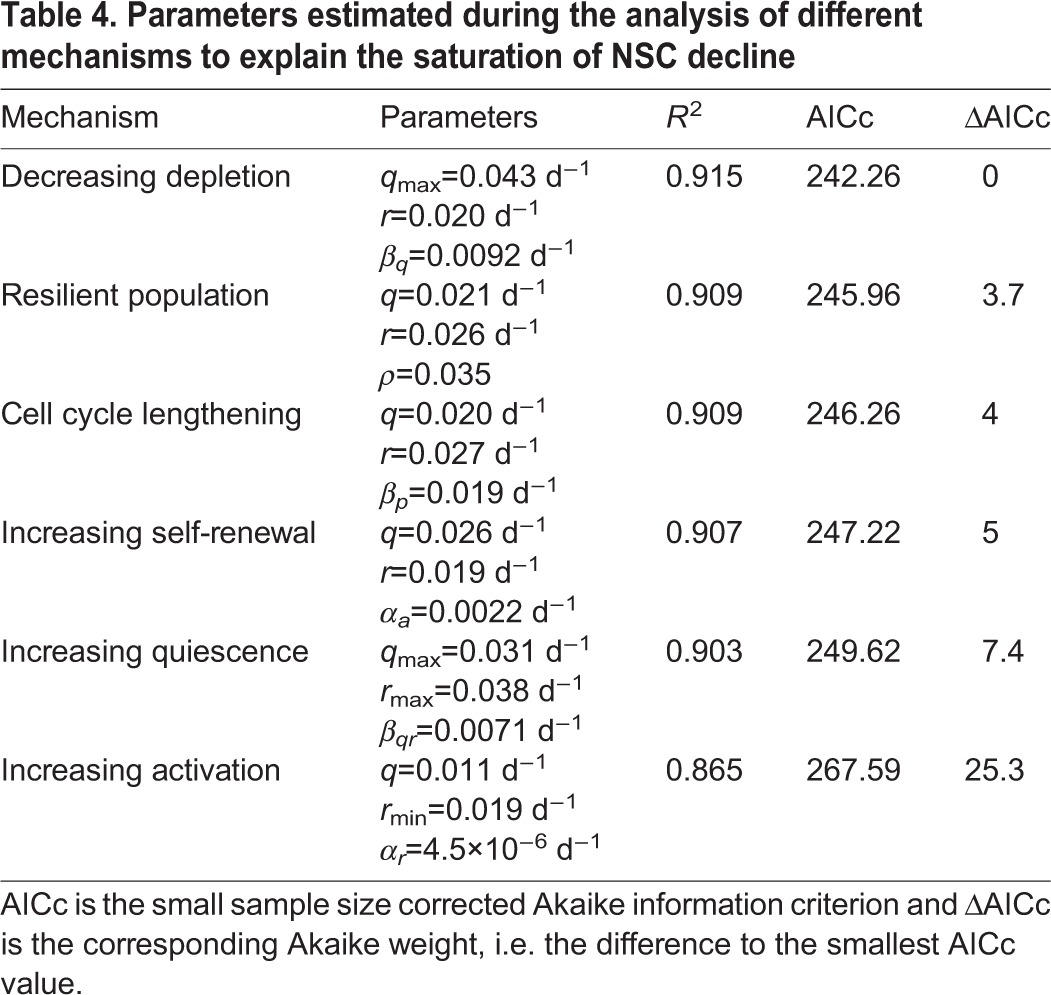
**Parameters estimated during the analysis of different mechanisms to explain the saturation of NSC decline**

To find the best explanation for the effects upon Dkk1 KO, we employ a nested approach by considering simple, i.e. few parameter-involving, explanations as well as more-complex scenarios (Table 5). At first, we assume that only one of the stem cell parameters *a*, *p*^stem^, *q*_max_ or *r* and, in addition, the neuroblast death rate *d* changes. The best fit to the data is achieved by a decrease of the depletion rate *q*_max_ or an increase of the activation rate *r*. In contrast, the scenario of increased self-renewal *a* displays a considerably worse fit. The decreased depletion and increased activation scenario both lead to a shift of the balance between NSC activation and depletion towards a higher fraction of activation events. We thus also consider a scenario in which both parameters *q*_max_ and *r* change simultaneously and find that it improves the fit. It is also possible that all NSC parameters change, resulting in an even better fit.

**Table 5.**
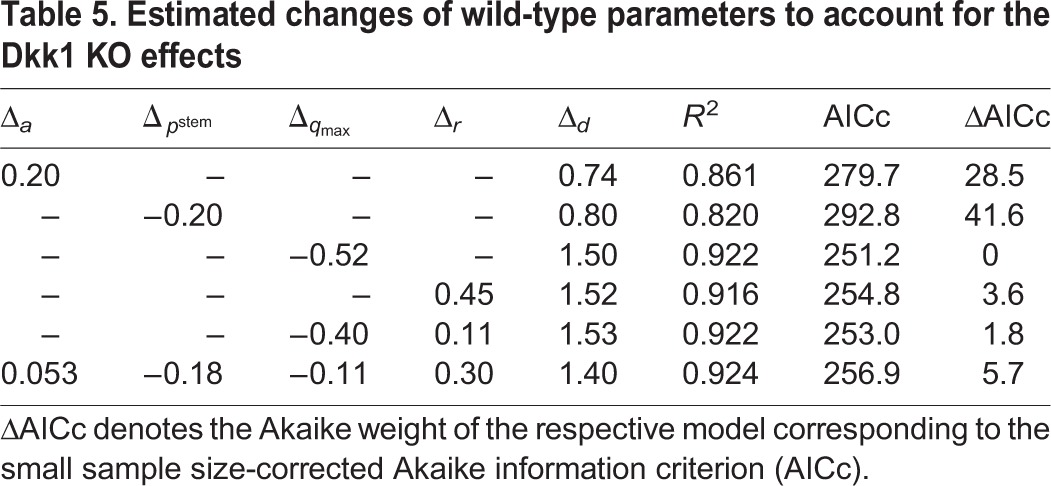
**Estimated changes of wild-type parameters to account for the Dkk1 KO effects**

Although the fit improves with an increasing number of parameters, we again computed AIC values to assess whether the increased goodness of fit justifies the additional complexity of the model. Applying the previously mentioned recommendation of 0≤Δ≤2, scenarios leading to the discussed shift of the balance between NSC activation and depletion towards a higher fraction of activation events should be considered first (Table 5). Moreover, the originally suggested increased self-renewal (Seib et al., 2013) of NSCs can be disregarded to explain the effects of Dkk1 deletion.

## Supplementary Material

Supplementary information

Supplementary information
