# Supplementary material for: Revealing age-related changes of adult hippocampal neurogenesis using mathematical models
Source: Development. 2018 Jan 1;145(1):dev153544. doi: 10.1242/dev.153544 (PMC5825879; doi:10.1242/dev.153544)
Supplement: Supplementary information [file develop-145-153544-s1.pdf]

Supplementary Information

S1 Stem Cell Depletion Coupled to Stem Cell Division

An alternative scenario for stem cell activation and reproduction process is based on a hypothesis that stem cell depletion takes place after a series of asymmetric division events and there is no return to quiescence(Encinas et al. 2011). This process can be described by the following system of equation

$$\begin{aligned}
\frac{\mathrm{d}}{\mathrm{d}t}c_0(t) &= -rc_0(t),\\
\frac{\mathrm{d}}{\mathrm{d}t}c_1^n(t) &= rc_0(t) - pc_1^n(t),\\
\frac{\mathrm{d}}{\mathrm{d}t}c_1^k(t) &= pc_1^{k+1}(t) - pc_1^k(t),\\
\frac{\mathrm{d}}{\mathrm{d}t}c_1^0(t) &= pc_1^1(t) - qc_1^0(t),
\end{aligned} \tag{S1.1}$$

where $n \in \mathbb{N}$ is the maximum number of asymmetric NSC divisions, c_0 denotes quiescent NSCs and c_1^k $(0 \leq k \leq n)$ cycling NSCs with k divisions remaining. The parameter r describes the activation rate of quiescent NSCs, p is the division rate of proliferating NSCs and q denotes the rate of transformation to astrocytes of NSCs that had already divided k times (Supplementary Information Fig. S1). Following the hypothesis of Encinas et al. (2011), we assume $n = 3$. Using the available proliferation rate measurements (4.1) allow to fit separately the model to different data sets (see Supplementary Information Figs. S2a & S2b).

To validate the models, we compare the dynamics of the clonal fit to the time-varying composition of the NSC pool. Model (S1.1) predicts that the fraction of cycling NSCs vanishes within three months (Supplementary Information Fig. S3a), what contradicts the constant fraction of BrdU incorporating NSCs during aging as observed in the experiments (Encinas et al. (2011) and Fig. 2f). In contrast, model (2.1) is in line with the data showing a constant fraction of proliferating stem cells (Supplementary Information Fig. S3b).

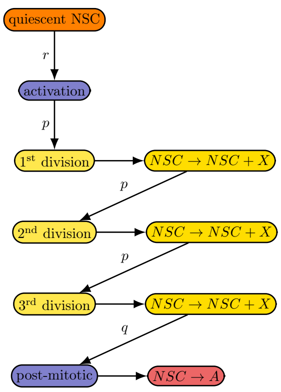

Figure S1: **Graphical representation of model** (S1.1)**.**
Quiescent NSCs can become activated to enter the cell cycle and subsequently perform a series of three asymmetric divisions by producing a NSC and another cell (X) before entering the post-mitotic stage and transforming into an astrocyte (A).

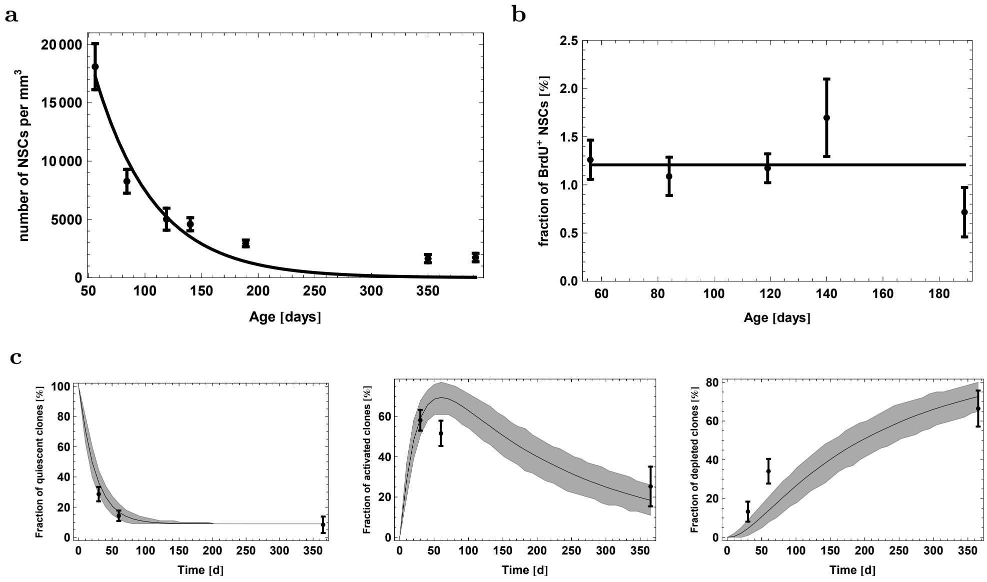

Figure S2: **Fit of model** (S1.1) **to population-level and clonal data.**
(**a**) & (**b**) Fit of the model to population-level data displayed in Fig. 2. Estimated parameters are $q = 0.030\,\mathrm{d}^{-1}$ and $r = 0.019\,\mathrm{d}^{-1}$
(**c**) Fit of the model to the clonal data of Bonaguidi et al. (2011). Results are obtained by simulating 100 NSC clones for 1000 times. Simulation data are represented as mean (solid black line) and (gray) band containing 95% of all simulated trajectories. Black error bars correspond to the clonal data. Estimated parameters are $q = 0.0048\,\mathrm{d}^{-1}$, $r = 0.042\,\mathrm{d}^{-1}$ and $\rho = 0.087$.

Development • Supplementary information

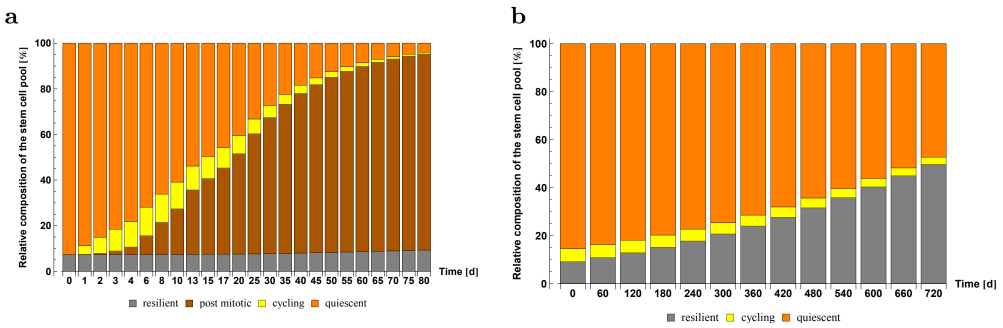

Figure S3: **Comparison of clonal dynamics of models** (S1.1) **and (2.1)**
Time course of the composition of the NSC pool in model (S1.1) (**a**) and model (2.1) (**b**) with the dynamics of the respective clonal fit. X-axis represents time in days, Y-axis relative amount in per cent.

Development • Supplementary information

S2 Astrocytic Transformation

We want to give an explanation for the claim that NSC apoptosis is almost non-detectable in the case of $1-\theta = 52.7\%$ of the NSC decline resulting from apoptosis. Consider the stem cell model (2.1) including aging effects (2.2). To model the dynamics of NSC apoptosis, we add a new compartment c_{A} accounting for *apoptotic*, i.e. biologically dead but physically present stem cells. The dynamics of NSCs at a certain age τ is thus given by

$$\begin{aligned}
\frac{\mathrm{d}}{\mathrm{d}t}c_0(t) &= -(r+q(\tau))c_0(t) + 2apc_1(t),\\
\frac{\mathrm{d}}{\mathrm{d}t}c_1(t) &= rc_0(t) - pc_1(t),\\
\frac{\mathrm{d}}{\mathrm{d}t}c_{\mathrm{A}}(t) &= (1-\theta)q(\tau)c_0(t) - \delta_{\mathrm{phag}}c_{\mathrm{A}}(t),
\end{aligned}$$

where δ_{phag} is the rate at which apoptotic cells are cleared via phagocytosis within, on average, $1/\delta_{\mathrm{phag}} = 1.5\,\mathrm{h}$ (Sierra et al. 2010). At the age of $\tau = 2$ months, there are about $n_0 = 10000$ NSCs in the entire dentate gyrus (Encinas et al. 2011) and the number of apoptotic NSCs can be calculated by that number times the steady state fraction of apoptotic NSCs on all NSCs. Accordingly, there are

$$n_0 \cdot \lim_{t\to\infty} \frac{c_{\mathrm{A}}(t)}{c_0(t)+c_1(t)+c_{\mathrm{A}}(t)} = 8$$

apoptotic stem cells in the entire dentate gyrus. Considering the usual sampling fraction of one sixth of the DG, there only remain about two apoptotic stem cells to be detected.

To analyze the scenario that the accumulation of astrocytes could be explained with a higher transformation rate θ if in addition astrocytes are allowed to undergo apoptosis, we consider the modified dynamics

$$\frac{\mathrm{d}}{\mathrm{d}t}c_2(t) = \theta q(t)c_0(t) + (1-\kappa)2(1-a)pc_1(t) - d_2c_2(t),$$

where d_2 is the death rate of astrocytes. Estimating θ and d_2 simultaneously yields $\theta = 0.364$ and $d_2 = 1.4\times10^{-5}\,\mathrm{d}^{-1}$, showing that there is no justification for such scenario. In addition, assuming $\theta = 1$ and only estimating d_2 results in an AICc score of 373.1, which compared to the AICc of 353.3 for the no-apoptosis model further indicates that there is no support for this scenario from a model selection viewpoint.

S3 Dynamics of Progenitor Cells

To model the dynamics of progenitor cells, we again make use of the study of Encinas et al. (2011). We consider the data set in which the authors label dividing cells with BrdU and track the number of BrdU labeled progenitors (Supplementary Information Fig. S4). Because of the rapid increase and subsequent decrease of labeled cells, they concluded that progenitors perform a series of symmetric self-renewing divisions, followed by subsequent transformation into neuroblasts.

Development • Supplementary information

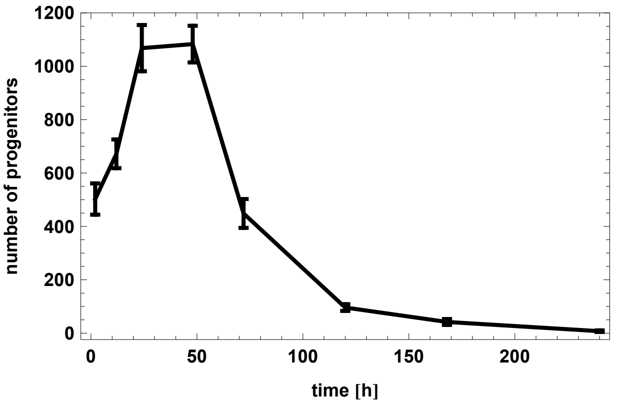

Figure S4: Time course of the dynamics of neural progenitor cells. Two months old mice were injected with BrdU and sacrificed at several time points after injection. Depicted is the number of BrdU positive progenitor cells. Data is reproduced from the publication of Encinas et al. (2011).

We model the dynamics of progenitors with the equations

$$\begin{aligned} \frac{\mathrm{d}}{\mathrm{d}t}P_k(t) &= 2pP_{k+1}(t) - pP_k(t), \\ \frac{\mathrm{d}}{\mathrm{d}t}P_N(t) &= -pP_N(t) \end{aligned} \tag{S3.1}$$

for $k = 0, \ldots, N-1$. Here, P_i is the number of progenitor cells with i remaining divisions and $p > 0$ is the proliferation rate. Moreover, we assume that at the start of the experiment, all progenitors have N remaining divisions, i.e. $P_N(0) = n$ for some $n > 0$ and $P_k(0) = 0$ for $k \neq N$.

For quantifying p, we consider the corresponding cell cycle length t_c, which is linked to p via (4.1). The cell cycle length of progenitor cells has been measured in different studies, however with contrasting results ranging from 12−14 h to about 22 h (Hayes and Nowakowski 2002; Farioli-Vecchioli et al. 2014). We thus employ an unbiased approach for quantification by assuming different cell cycle lengths t_c and compute the R^2 of the fit dependent on N, the maximum number of progenitor divisions (Supplementary Information Fig. S5).

The best fit can be obtained for $N = 2$, 3 or 4, but only $N = 2$ allows for a cell cycle length in the range of what is experimentally observed with the maximum R^2 at $t_c = 14.4$ h. To achieve a better compromise between our model assumption and the measured cell cycle lengths, we relax the condition of an optimal R^2. A visual assessment of the fit shows that $R^2 = 0.95$ provides a reasonable fit to the data. For $N = 2$, the maximal t_c for which $R^2 = 0.95$ can be achieved is $t_c = 15.6$ h (Supplementary Information Fig. S6), which we assume for our subsequent analysis.

Development • Supplementary information

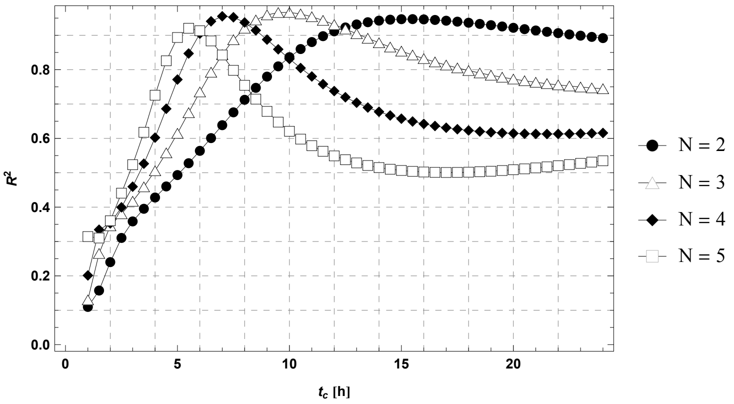

Figure S5: Goodness of fit of the progenitor model (S3.1) to the data displayed in Supplementary Information Fig. S4. The R^2 is calculated solely from the first five time points of the data, which capture the rapid rise and fall of progenitor numbers.

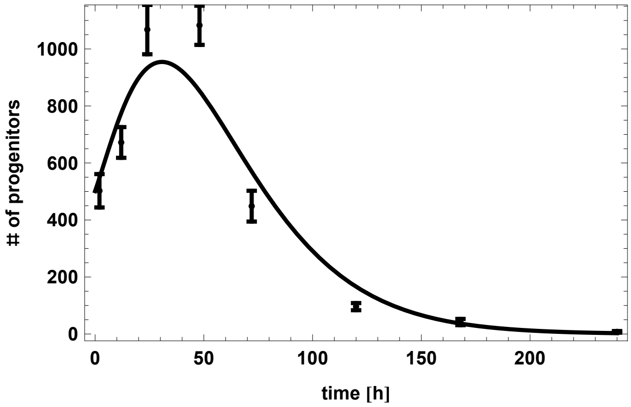

Figure S6: Fit of the progenitor model (S3.1) to the data of Supplementary Information Fig. S4, assuming $N = 2$ progenitor divisions and a cell cycle length of $t_c = 15.6\,\mathrm{h}$.

Development • Supplementary information

S4 Sensitivity Analysis

Model (2.1) contains three unknown parameters (a, q and r), which needed to be estimated from the data. It is interesting to analyze how variability in those parameters affects downstream findings that depend on estimated values. We investigate two questions. First, we check whether the clonal data of Fig. 3 can be fitted using the population-level estimates of model (2.1) by allowing only one parameter to vary. This would indicate that uncertainty in the estimate of one parameter could indeed explain population-level and clonal dynamics simultaneously. Second, how uncertainty in the population-level estimates of model (2.1) transfers to the analysis of different scenarios to explain the saturating decline of NSC numbers during aging.

S4.1 Clonal Fit with One Varying Population-Level Parameter

We fit the clonal data as outlined in the Materials and Methods section using the population-level estimates of parameters q and r shown in Table 1 and the previously stated assumption $a = 0.525$. Changing parameter q results in a rapid decline of the fraction of quiescent clones (Supplementary Information Fig. S7a). Estimating r leads to a model fit with a fraction of activated and depleted clones, which does not match the one year time point data (Supplementary Information Fig. S7b). Finally, if only parameter a varies, the one year time point cannot be matched for any of the three time series (Supplementary Information Fig. S7c).

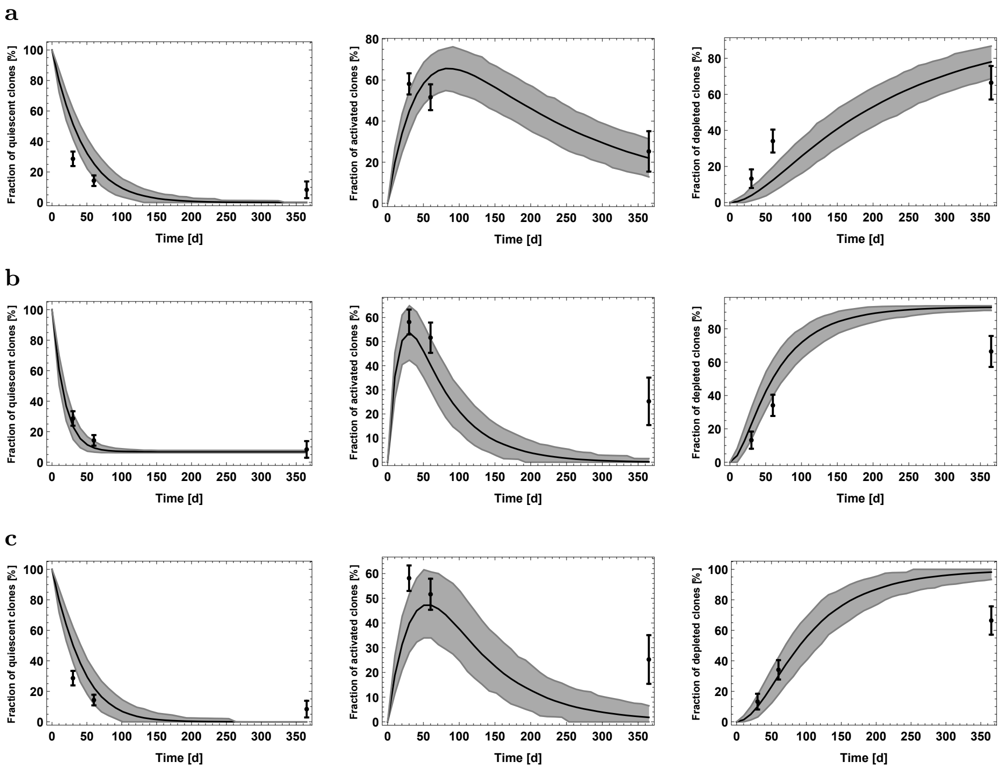

Figure S7: Fit of model (2.1) to the clonal data of Bonaguidi et al. (2011) using population-level estimates of parameters but allowing one parameter to vary. S7a: Parameter q is estimated ($q = 0.0055\,\mathrm{d}^{-1}$, $\rho = 8.3 \times 10^{-4}$). S7b: Parameter r is estimated ($r = 0.047\,\mathrm{d}^{-1}$, $\rho = 0.053$). S7c: Parameter a is estimated ($a = 0.70$, $\rho = 0.0028$).

Development • Supplementary information

S4.2 Saturating Stem Cell Decline

Validating the models accounting for different scenarios to explain the saturation of the decline of NSC numbers, we re-estimate the parameters q and r (Table 4). Hence, model findings are independent of the original population-level estimates. However, findings depend on the assumption of 5% of stem cell divisions being symmetric, i.e. $a = 0.525$. To investigate the sensitivity of the results in respect to this assumption, we re-estimate parameters of all age-related scenarios, assuming either a lower self-renewal rate ($a = 0.5$) or a higher one ($a = 0.6$). As can be seen from Supplementary Information Table S1, such variation of the self-renewal fraction does not change the results of model selection. The alternative scenarios remain to have at least 2.5 points higher AICc score.

Development • Supplementary information

Table S1: AICc scores of fitting all considered mechanisms to explain the decline pattern of NSCs numbers, assuming a lower or higher fraction of self-renewal a, compared to the default assumption $a = 0.525$.

a	Mechanism	AICc
0.5	Decreasing depletion	241.967
0.5	Resilient population	245.711
0.5	Increasing self-renewal	246.332
0.5	Cell cycle lengthening	246.496
0.5	Increasing quiescence	249.881
0.5	Increasing activation	267.598
0.525	Decreasing depletion	242.26
0.525	Resilient population	245.96
0.525	Cell cycle lengthening	246.26
0.525	Increasing self-renewal	247.22
0.525	Increasing quiescence	249.62
0.525	Increasing activation	267.59
0.6	Decreasing depletion	243.039
0.6	Increasing self-renewal	245.553
0.6	Resilient population	245.754
0.6	Cell cycle lengthening	248.219
0.6	Increasing quiescence	250.446
0.6	Increasing activation	266.024

Development • Supplementary information
